# Irradiation-Equivalent Injection Test Method for Analyzing HEMP Coupling Effects on Communication Interfaces

**DOI:** 10.3390/mi17101205

**Published:** 2026-10-20

**Authors:** Lu Sun, Peihong Wu, Li Yang, Jin Tian, Zhenxiao Li, Feifei He, Yang Qiu

**Affiliations:** State Key Laboratory of Electromechanical Integrated Manufacturing of High-Performance Electronic Equipment, School of Electro-Mechanical Engineering, Xidian University, Xi’an 710071, China; 24041212777@stu.xidian.edu.cn (P.W.); 13659268022@139.com (Z.L.); 18165195224@139.com (F.H.); qiuyang@vip.sina.com (Y.Q.)

**Keywords:** communication interface, high-altitude nuclear electromagnetic pulse, injection tests, damage assessment

## Abstract

The E1 component of High-altitude Nuclear Electromagnetic Pulse (HEMP) is an electromagnetic wave with extremely high peak power and fast-rising transient fields that can cause irreversible damage to electronic devices in an extremely short period of time. Traditional irradiation tests used to assess its impact on communication interfaces have problems such as high costs and complex system setup. Moreover, HEMP injection test standards and the actual coupling waveforms of the interfaces have significant differences, which leads to inaccurate electromagnetic coupling effect test results and causes problems through unreasonable design of the protection circuit. This study, in combination with the field-line coupling simulation analysis of communication interface circuits, designs a coupling effect test method for irradiation-equivalent injection without the need for high-cost irradiation equipment. By setting up a test system to conduct equivalent injection tests, taking RS232 communication interfaces as a typical representative, the interference threshold of the interface circuits can be accurately obtained. This enables the implementation of targeted and accurate protective measures to ensure the safe use of electronic devices, and has significant engineering application value.

## 1. Introduction

Communication interfaces play a crucial role in high-speed data transmission and interaction in modern electronic systems. They are usually directly connected to external devices or communication networks, thus they are exposed to a relatively high risk of electromagnetic interference. Especially in modern communication systems, many interface circuits adopt high-speed and high-density integrated circuit designs. Although such designs can enhance system performance, they also mean that the anti-interference capability of interface circuits is relatively weak. Their sensitivity to HEMP can cause system-level failures and even threaten the safety of critical infrastructure. The transient strong electromagnetic fields of HEMP with nanosecond rise times and field strengths of tens of kV/m can inject interference through cable coupling and spatial radiation [1,2,3], causing faults such as data packet loss, protocol collapse, and chip breakdown in interface circuits. Typical interference phenomena [4,5,6] include communication interruption, video signal distortion or permanent damage to equipment. Such effects are particularly detrimental in high-risk scenarios such as military communications, aerospace, and power control. Therefore, analyzing and obtaining the HEMP coupling effects on communication interfaces accurately and providing effective protection is of great significance for the efficient operation of modern electronic systems.

Research on the strong electromagnetic effects of devices, equipment, or systems can be divided into two types, including research on strong electromagnetic pulse irradiation effects and that on injection effects [7,8,9]. Among them, the research on strong electromagnetic pulse irradiation coupling effects mostly relies on actual irradiation tests to determine failure thresholds and propose corresponding protective measures. This test method replicates the actual on-site environment. However, irradiation method test equipment is complex, costly, and difficult to build, making it difficult to efficiently repeat experiments and resulting in poor consistency in experimental results. Therefore, electromagnetic pulse injection experiments are often conducted to simulate situations where external electromagnetic waves couple into the equipment through conductors. However, in the application of HEMP protection, the injection test standard MIL-STD-188-125 [10] only uses the coupling current of the HEMP double-exponential waveform injected into electronic equipment as the basis for protection, which has a significant difference from the coupling waveform of interface cables in real irradiation scenarios. This makes it inaccurate to analyze the HEMP coupling effect and provide effective references for subsequent protective circuit design. Therefore, this study uses field-line coupling simulation of HEMP on communication cables to obtain accurate coupling waveform on the cables, reproduces this with an attenuated oscillation wave signal source, and directly injects it into communication interface circuits to obtain the coupling waveform characteristics on the communication interfaces [11]. This test method, compared to traditional HEMP injection waveform coupling results, can more accurately represent the interference and damage conditions of the interfaces and provide effective references for the design of protective circuits.

It should be noted that damped sinusoidal injection has already been specified in MIL-STD-461G [12] and IEC 61000-4-18. However, these standards only define the injection waveform itself, and do not specify, for a given communication interface board, which specific frequency and amplitude should be injected into this circuit board. In other words, the existing standards provide a general injection method, but not a coupling-effect-oriented injection condition for specific communication interfaces. In this work, for typical communication interfaces based on the actual coupling waveform obtained from field-line coupling simulation, the injection test is carried out under a waveform that is consistent with the actual coupling condition of the interface, and the focus is placed on the comparison of the coupling effects among different interfaces rather than on the injection method itself.

To highlight the contributions of this work more explicitly, the core advantages of the proposed scheme over traditional test methods are summarized in Table 1.

## 2. Time-Frequency Domain Characteristics of HEMP Irradiation Waveforms and Their Coupling Relationship with Communication Interfaces

The direct injection method cannot accurately restore the HEMP irradiation environment, which affects the accuracy of the coupling effect test results. The main reasons for its inaccuracy are the lack of accurate analysis and judgment of the complex propagation and coupling process of electromagnetic waves, as well as interface impedance mismatch. The disadvantage of the HEMP irradiation test is its high cost. This is mainly due to high test equipment standards, the difficulty in controlling the test environment, strict safety requirements, and the complexity of the test. The electric field waveform of the HEMP irradiation test is stipulated by MIL-STD-188-125 standards; the waveform requirements for HEMP irradiation have a rise time within 2.3 ± 0.5 ns and a half-width within 23 ± 5 ns, and the field strength standard of 50 kV/m ensures consistency and reliability. Due to the limitations of test instruments and test sites, it is currently difficult to conduct strong electromagnetic pulse irradiation tests on cables. At the same time, there are cases where the standard injection waveforms do not match the actual coupled waveforms. Therefore, the core aim of using this technology is to explore the real coupled waveforms of HEMP on communication cables, and on this basis, build an equivalent injection device of the real coupled waveforms, accurately study the coupling and damage effects of communication interconnection cables and interfaces in the HEMP environment, and reduce the complexity and cost of the experiment.

In the HEMP irradiation standard, its definition is a double-exponential waveform, which is presented as follows:

*E*(*t*) = *E*_0_*k*(*e*^−*αt*^ − *e*^−*βt*^) (1)

In the formula above, *E*_0_ represents the peak field strength of the pulse measured in V/m, k is the correction coefficient for the pulse amplitude, *α* is the attenuation coefficient of the pulse leading edge mainly affecting the rising edge, and *β* is the attenuation coefficient of the pulse trailing edge, mainly affecting the falling edge.

**Figure 1:**
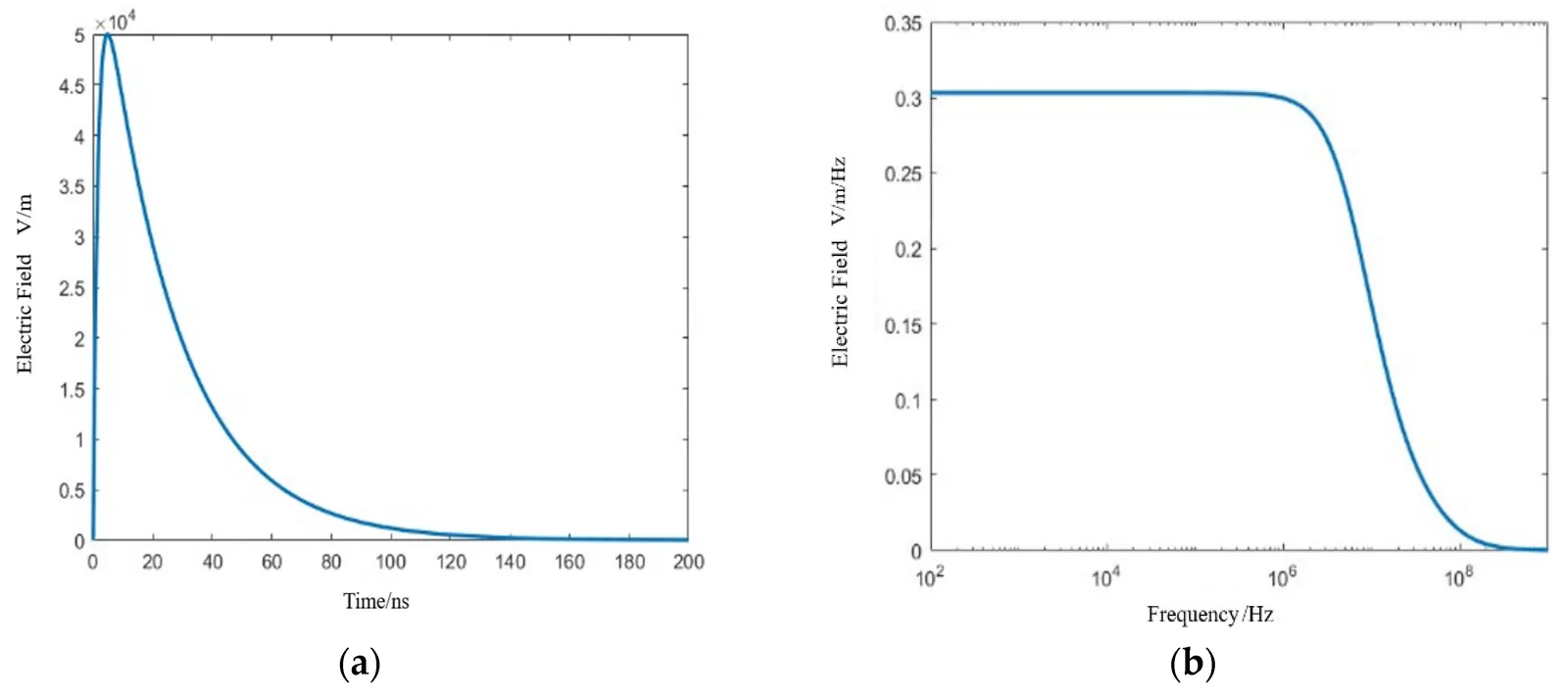
Time and frequency domain waveform of HEMP during irradiation: (**a**) time domain waveform. (**b**) Frequency domain waveform.

In MIL-STD-461G, the definitions of each parameter in the irradiation waveform function expression of HEMP are given. The values of *E*_0_ are determined as 50 kV/m, *k* as 1.3, *α* as 4 × 10^7^/s, and *β* as 6 × 10^8^/s. Substituting these values into the function expression yields the time-domain pulse waveform of this HEMP, and the frequency-domain pulse waveform can be obtained through FFT (Fast Fourier Transform) as shown in Figure 1.

At the same time, HEMP has extremely high peak field strength, which can generate a pulse response current in communication cables within an extremely short time and transfer it to the interface circuit of the communication equipment. Due to the coupling effect of the instantaneous current, the chips or components inside the equipment will rapidly heat up, causing the interface circuit to fail or be damaged, seriously threatening the safety of the communication equipment.

## 3. Acquisition and Implementation of HEMP Irradiation Coupled Waveform for Communication Cables

### 3.1. Design of Field-Line Coupling Model and Analysis of Coupled Waveform

To obtain the actual coupling wave of the communication interface under HEMP irradiation, it is necessary to first establish a field-line coupling model [13,14,15] between the HEMP irradiation and the communication cables and simulate the coupling effect of the communication interface boards under HEMP irradiation in normal communication conditions. In the simulation, the influence of different cable lengths and their different placement heights are considered. In this study, a computer is used as the communication system, and has multiple types of communication interfaces including RS232, RS485, USB, RJ45, and HDMI.

In an electromagnetic pulse environment, when the communication system is in a normal communication state, strong electromagnetic pulse irradiation will couple to the line through the connection point between the interface board and the cable. Using the plane wave irradiation method, it simulates the normal communication working state and the two ends of the computer interconnection cables are connected to communication interface boards.

**Figure 2:**
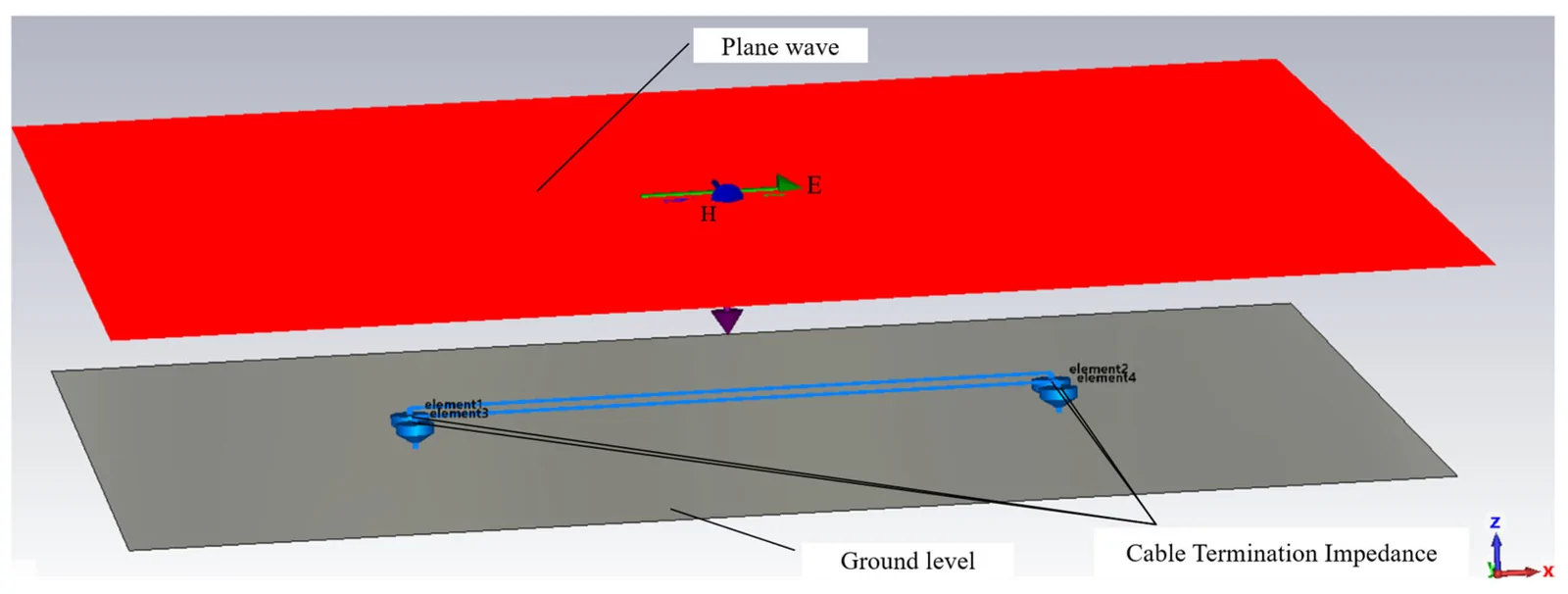
Interconnection cable field-line coupling model.

As shown in Figure 2, a field-line coupling model is built and the coupling model parameters are set as follows.

The field-line coupling simulation is carried out using CST (CST Studio Suite 2020) electromagnetic simulation software. The ground plane is defined as a Perfect Electric Conductor (PEC) plane, representing the reference ground in the actual test setup. The termination impedances at both ends of the cable are not ideal 50 Ω values; instead, they are set to the actual equivalent impedances of the communication interface boards under the HEMP coupling state, which are obtained by combining HEMP injection tests with PSPICE equivalent-circuit fitting. In this way, the simulation model reflects the real port impedance of the interface board rather than an idealized matched load.

The cable radius is set to 0.5 mm according to the common radius of computer interconnection cables, and the grounding impedance at both ends of different cables to the HEMP is the actual impedance in the normal working state, which can be obtained under real experimental conditions. We set the cable height from the ground to 0.01 m, and set the lengths as 0.1 m, 0.5 m, 1 m, 1.5 m, and 5 m.

The waveform of the HEMP irradiation source is set according to the MIL-STD-461G standard. The transmission line is parallel to the ground plane, and the excitation source uses a plane wave with normal incidence on the ground plane. When the plane wave is normally incident on the ground plane, the electric field is parallel to the transmission line, and the magnetic field is perpendicular to the plane formed by the transmission line and the ground plane. In this case, the electric field component along the transmission line reaches its maximum, and the induced voltage and current on the cable are also maximized. If the incident angle deviates from the normal direction, the effective electric field component parallel to the transmission line decreases, and the coupling efficiency is reduced accordingly. Therefore, normal incidence with the E-field parallel to the transmission line represents the most severe coupling condition, and this condition is adopted in the simulation to obtain the worst-case coupling current waveform.

Due to the various types of communication interfaces for electronic devices with different termination impedance, including RS232, RS485, and Ethernet and display interfaces, different cable coupling waveforms can result in attenuation oscillation waveforms of different frequencies and amplitudes.

**Figure 3:**
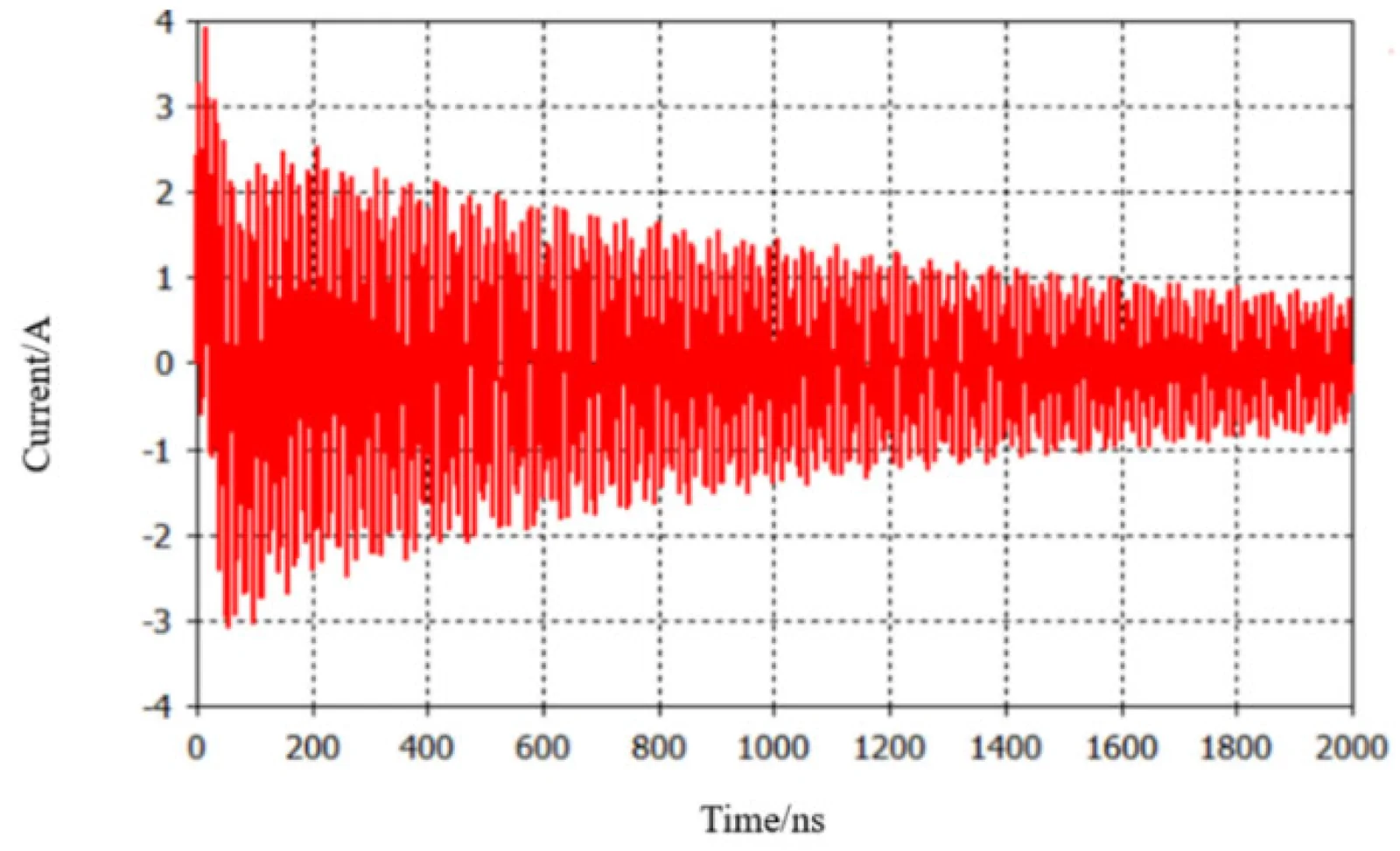
Current waveform of the RVSP line connection for the RS-232 communication interface, 0.1 m in length.

In Figure 2 and Figure 3, the key parameters of the field-line coupling model correspond to the actual experimental test apparatus. Specifically, the cable radius is set to 0.5 mm according to the radius of the common computer interconnection cables; the cable height above the ground is set to 0.01 m, which is consistent with the cable layout height adopted in the actual injection test; and the cable lengths are set to 0.1 m, 0.5 m, 1 m, 1.5 m and 5 m so as to cover the typical interconnection lengths of the communication interfaces. More importantly, the termination impedances at both ends of the cable are not the ideal value of 50 Ω, but the actual equivalent impedances of the communication interface boards under the HEMP coupling state. Therefore, the field-line coupling model in Figure 2 and the coupling current waveform in Figure 3 are not purely idealized simulation results, but are established on the basis of the real cable geometry, the real layout height, and the measured port impedances of the interface boards.

Based on the field-line coupling simulation results, the injection test conditions are further displayed in Table 2. According to the simulated coupling current frequencies of the RS-232 interface with different cable lengths, and considering the actual data transmission application of the communication interface board, the cable length of 1 m, the above-ground cable height of 0.01 m, and the damped oscillating wave frequency of 84 MHz are finally selected for the injection test. In the present study, the injection test is carried out only for the RS-232 interface as a representative, so as to verify the consistency between the simulation model and the actual measurement under the condition that the cable length, cable height and port impedance are all consistent with the actual test setup.

For RS-232, RS-485, VGA and HDMI interfaces, as the cable length increases, the frequency of the coupling current gradually decreases. For the RS-232 interface, at a 0.1 m cable length, the coupling current frequency is 289 MHz, while at a 1.5 m cable length, it drops to 69 MHz. This frequency reduction is mainly due to the changes in the distributed capacitance and inductance of the cable as the length increases, resulting in a decrease in the resonant frequency. The 100-Megabit Ethernet interface, due to its built-in network isolation transformer, has a significantly lower coupling current frequency than other interfaces. At a 0.1 m cable length, the coupling current frequency of 100-Megabit Ethernet is only 19 MHz, much lower than that of other interfaces. This indicates that the network isolation transformer plays an important role in suppressing high-frequency coupling currents.

For shorter cables of 0.1 m and 0.5 m, the coupling current waveform shows a regular attenuation oscillation wave, with a high frequency and a clear peak. This indicates that in short-distance transmission, the impedance characteristics of the cable are relatively stable, and the coupling current is mainly determined by the distributed parameters of the cable. As the cable length increases, the coupling current waveform gradually becomes irregular, with an uncertain period, and even shows stray waves. This phenomenon may be due to the changes in the resistance, capacitance and inductance parameters of the cable as the length increases, which in turn causes a significant fluctuation in the AC impedance.

From the above field-line coupling results, it can be seen that under the HEMP irradiation condition, the waveforms actually coupled to the communication interface cable are bipolar characteristics of attenuation oscillation waveforms of different frequencies and peaks, which are significantly different from the equivalent injected HEMP waveforms of 20/500 ns stipulated by the MIL-STD-461G standard. Therefore, for the analysis of the coupling effect of communication interfaces, how to construct a real injection test condition for the communication interface circuit and accurately test and evaluate its coupling effect is of great significance for the subsequent protection design.

### 3.2. Generation of Attenuated Oscillation Source

Based on the waveform characteristics of the HEMP coupling [16,17,18] on the communication cable, without any protection, the waveforms in the cable will enter the electronic device through the interface. Under certain intensities, this will cause interference in the signal transmission of the electronic device and could even damage the electronic device. Therefore, it is extremely important to generate an equivalent interference source in the form of this attenuation oscillation and to accurately couple it with the communication interface.

The generation principle of the attenuated oscillation wave can be introduced as follows: the dynamic circuit described by a second-order differential equation is a second-order circuit, among which the RLC series circuit is one of the simplest second-order circuits. In the RLC series circuit, a process of attenuated oscillation discharge occurs when the damping coefficient is *ζ* < 1, and the damping coefficient expression is as shown in Equation (2):

(2) $$\xi =\frac{R}{2\sqrt{\frac{L}{C}}}$$

The magnitude of the damping coefficient reflects the speed of energy loss in the system. The greater the damping coefficient, the faster the energy loss, and the faster the vibration attenuation. Specifically, when the system is in an underdamped state, the circuit will undergo oscillation, and the amplitude of the oscillation will gradually decrease over time. The attenuation rate and the oscillation frequency both depend on the magnitude of the damping coefficient. Therefore, by adjusting the values of the resistors, inductors, and capacitors, the damping characteristics of the circuit can be controlled.

**Figure 4:**
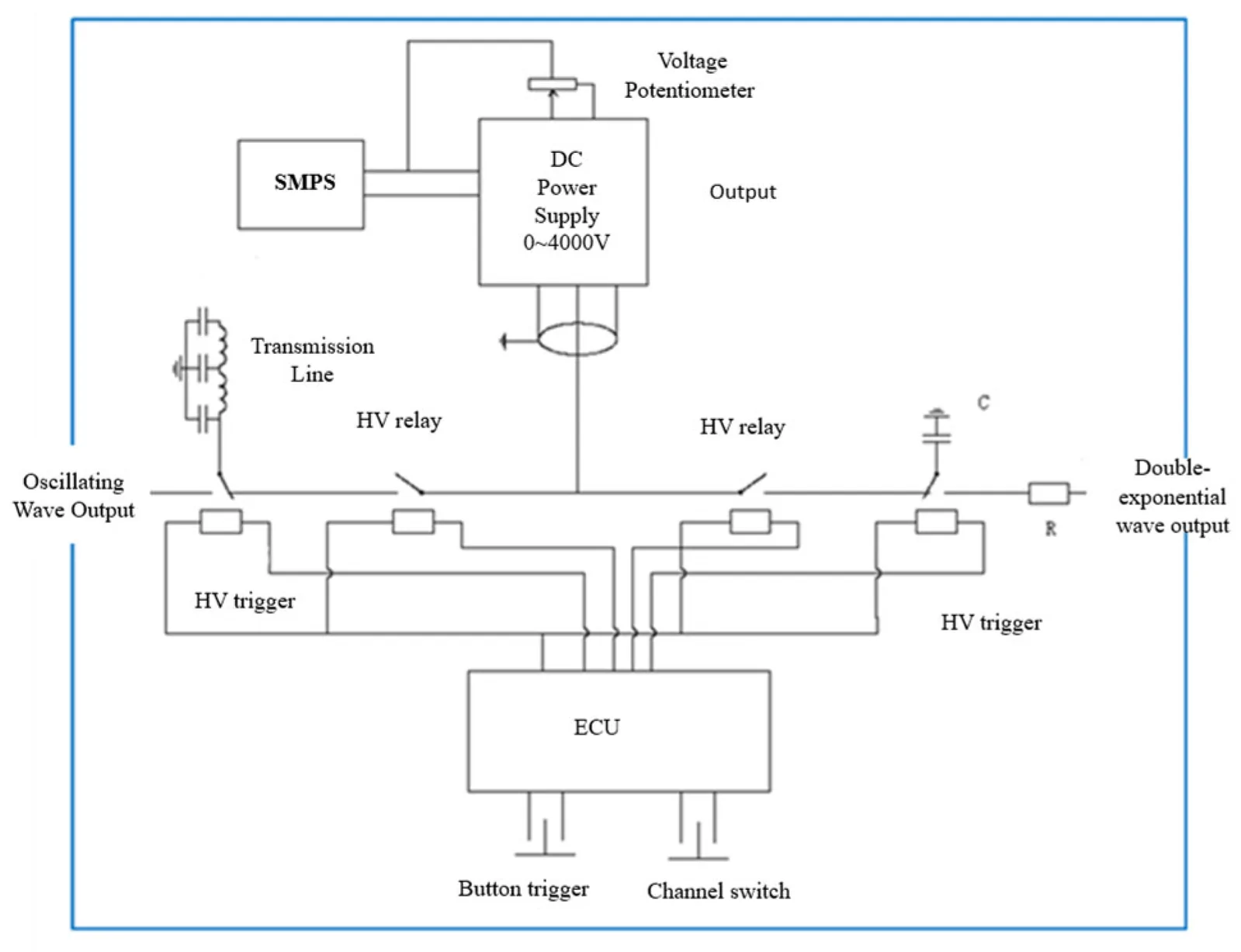
Pulse current injection source circuit.

**Figure 5:**
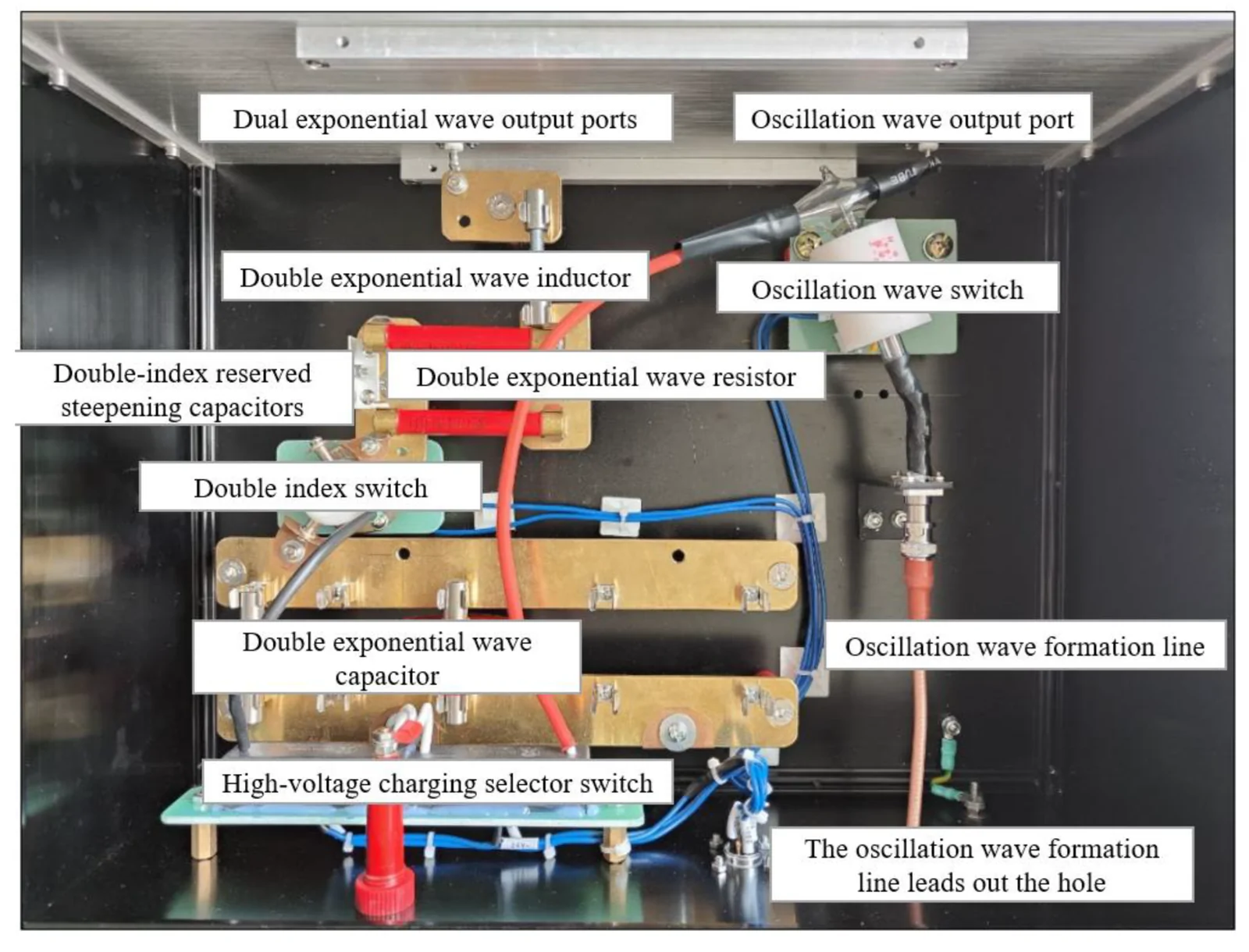
Internal composition diagram of the main circuit box of the pulse current injection source.

The main circuit of the attenuated oscillation wave pulse current injection source uses a high-voltage transmission line as the pulse transmission line. The transmission line can be equivalent to that of a model of multiple LC networks in series, and the DC power supply charges the LC network body. The more LC network levels, the more accurate the simulation model, and the smoother the output waveform. The characteristic impedance of the transmission line is selected as a 50 Ω coaxial cable, as shown in Figure 4, to form a line simulation circuit that can generate the attenuated oscillation wave output waveform. The internal composition diagram of the main circuit box is shown in Figure 5. 

After the DC power supply charges the LC network step by step, the switch is triggered to close, and the charge releases along the transmission line, forming a high-frequency attenuated oscillation wave. The high-voltage coaxial cable is used as the transmission line, and its physical length can be adjusted to control the frequency of the attenuated oscillation wave. Inside the main circuit, the oscillation wave output port and the double-exponential wave output port operate independently, and the HV (high-voltage) charging line supplies energy. The high-voltage charging selection switch selects different circuits, and the trigger switch controls the pulse release to ensure the generation of a stable high-frequency attenuated oscillation wave. An SMPS (switched-mode power supply) and an ECU (Electronic Control Unit) can provide an energy source and control unit.

Finally, based on the actual data transmission application of the communication interface circuits, the cable lengths between various communication interface boards and the frequency of the injected attenuation oscillation wave pulses are determined, as shown in Table 3. They can serve as a reference for the actual injection into the communication interface circuit.

## 4. Design of the Strong Electromagnetic Pulse Effect Test System and Analysis of Test Results

**Figure 6:**
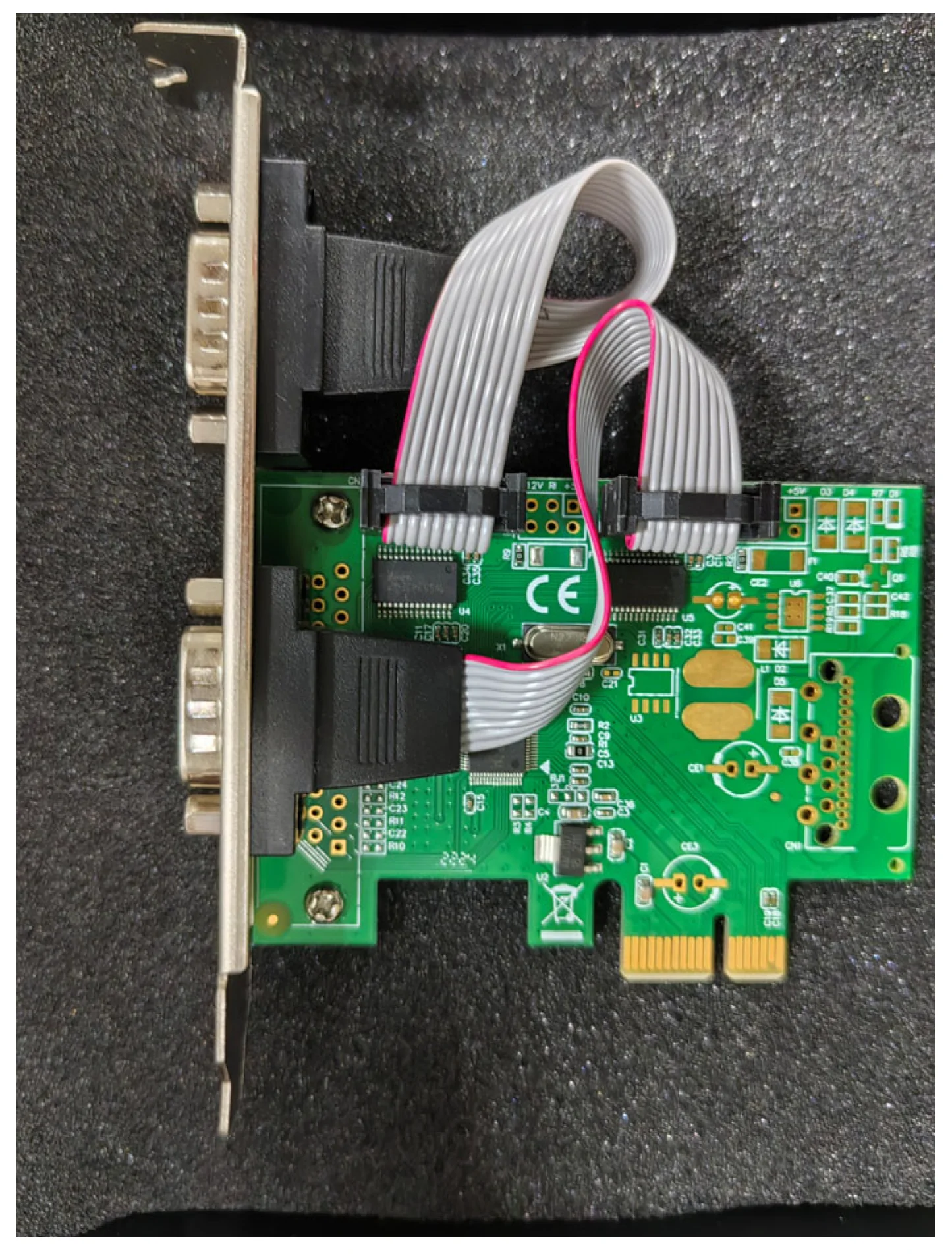
RS-232 communication interface board used in this test.

**Figure 7:**
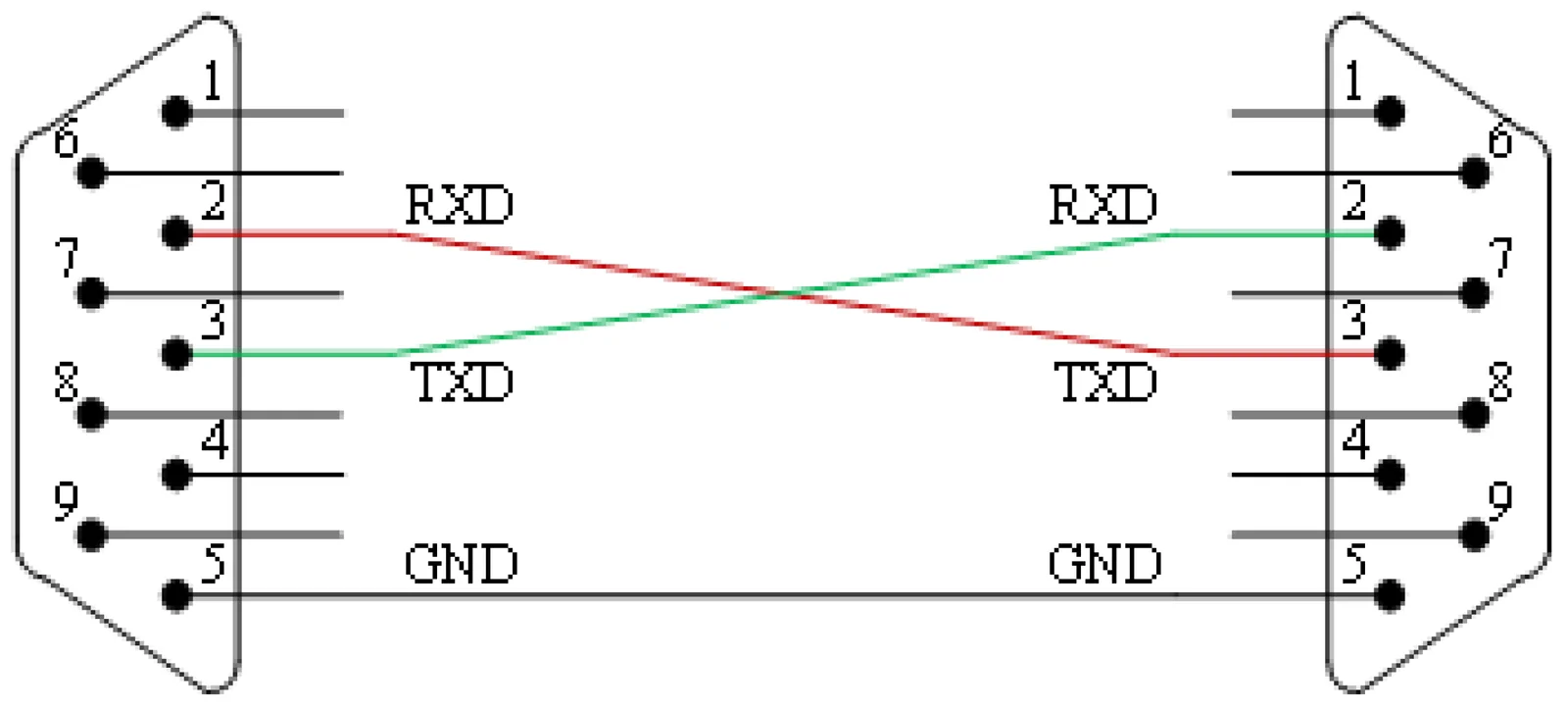
Interconnection diagram for three-wire RS-232 interface communication.

**Figure 8:**
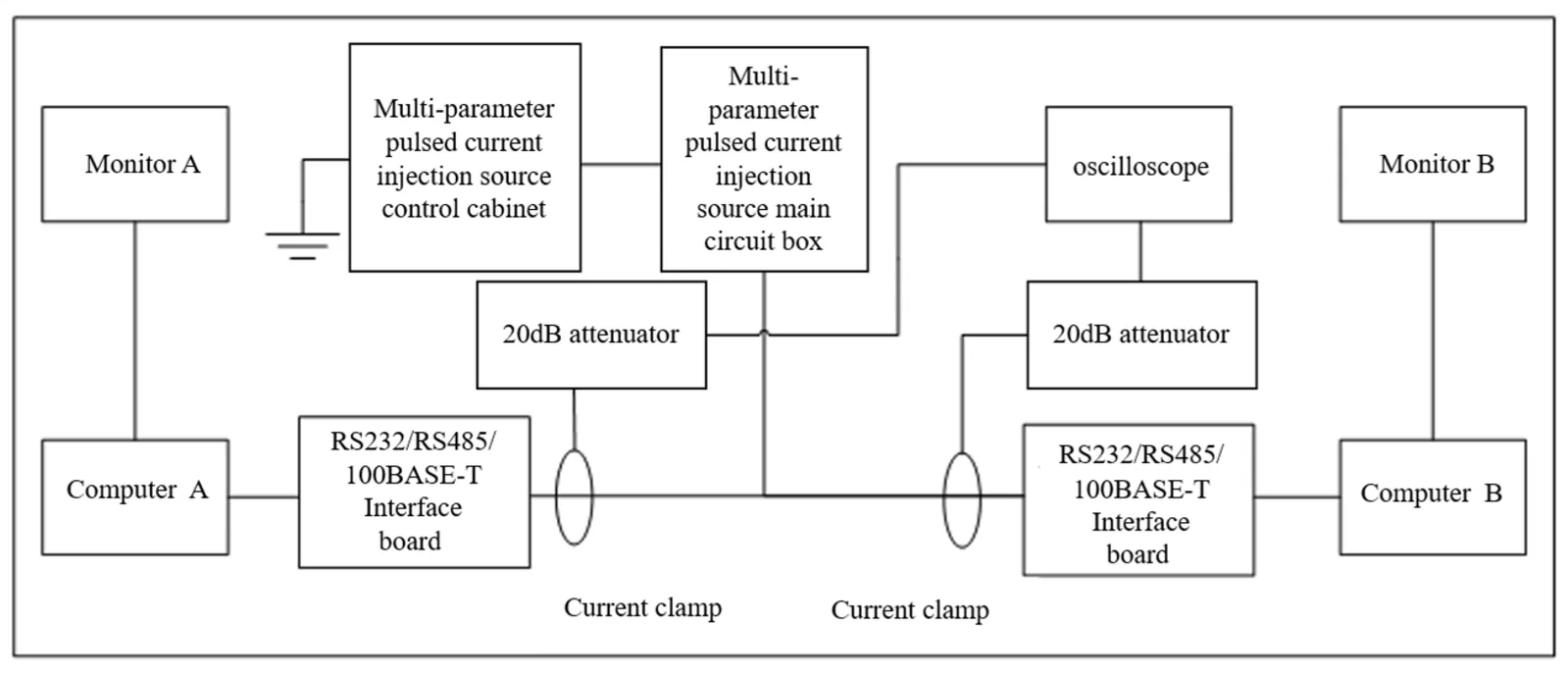
Test platform for the strong electromagnetic pulse effect of low-speed communication interface circuits.

**Figure 9:**
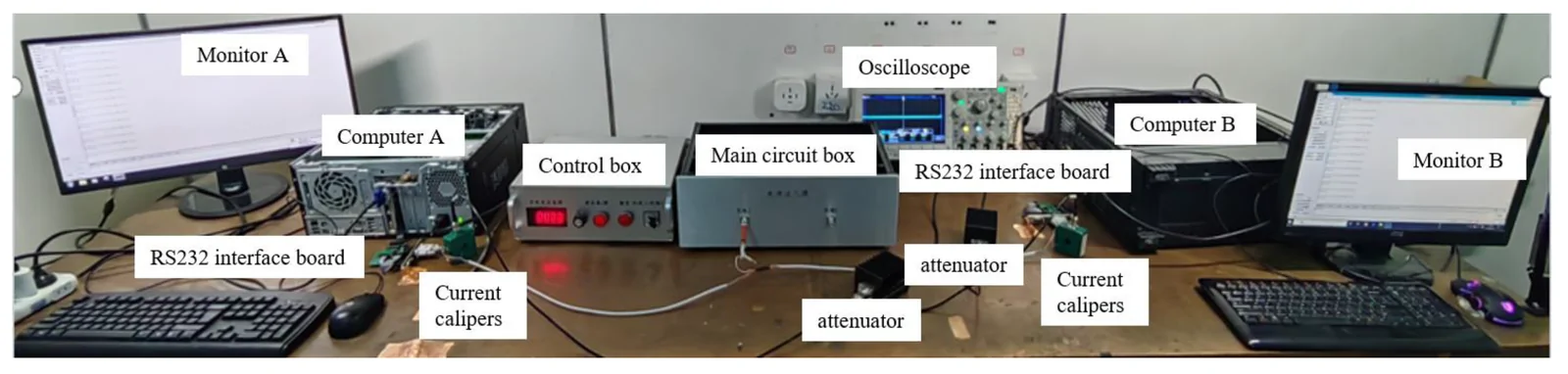
Effect test diagram of strong electromagnetic pulse injection computer interface.

Different communication interfaces will undergo injection tests on the platform for strong electromagnetic pulse effects in low-to-medium-speed communication interface circuits. During the injection tests, the focus is on the stability and anti-interference ability [19,20,21] of data transmission between the computer and the communication interface, specifically reflected in the context of data sending and receiving in the software window of the serial port/network port debugging assistant. After one type of communication interface board card test is completed, only the replacement of the communication interface board card and its communication cable type are required to conduct the next group of tests. Here, the RS-232 communication interface boards with two DB-9 ports are chosen and are shown in Figure 6, and the interconnection diagram is shown in Figure 7. When in use, two boards need to be used as the sending and receiving ends respectively. Figure 8 and Figure 9 present the principal diagram and effect test diagram of the strong electromagnetic pulse effect test platform for low-to-medium-speed communication interface circuits.

**Figure 10:**
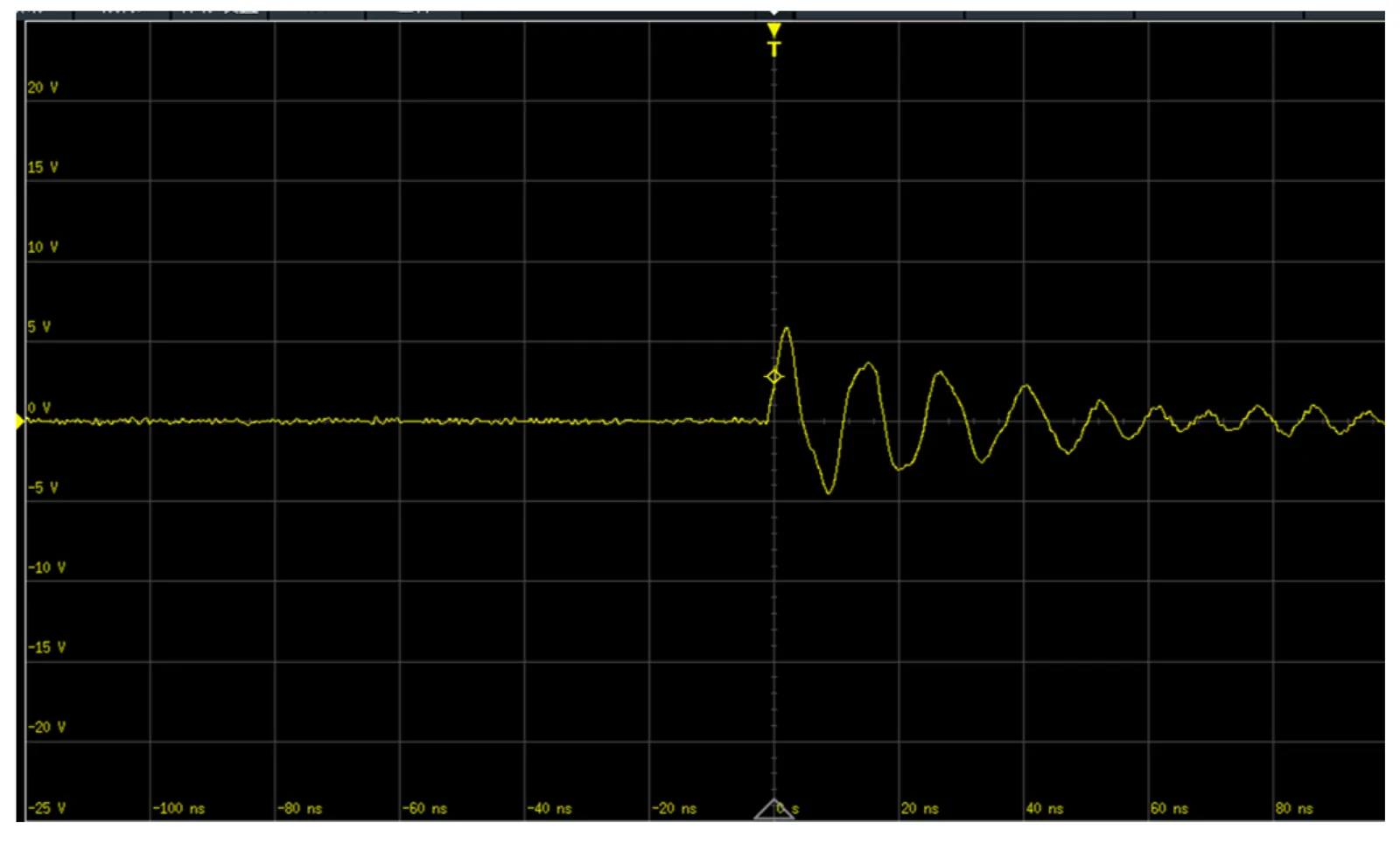
Attenuated oscillation wave injected in the RS-232 interface from the pulse current source.

During the test, open the serial port debugging assistant software (UartAssist V5.0.2) for both Computer A and Computer B, and configure the communication baud rate to 9600 bps. Computer A sends hexadecimal data 0X55, 0X56, and 0X57 at a 100 ms interval in a loop, and computer B is responsible for receiving the data. Set the attenuation oscillation wave pulse applied by the pulse current injection source to 84 MHz by changing the length of the transmission line shown in Figure 4, and ensuring that the signal can be correctly injected into the test system. The attenuated oscillation wave applied by the pulse current injected from the source as shown in Figure 10.

At the same time, observe the data sending and receiving activity in the serial port debugging assistant software windows on display A and display B with the increase in injection voltage.

If both computers send and receive data normally or if there are temporary faults that can be automatically recovered, which can be considered the interference, then inject an 84 MHz attenuation oscillation wave pulse with a step of 100 V. If both computers have faults where sent or received data cannot be automatically recovered, then the peak value of the coupling current at this time is determined to be the damage threshold of the RS-232 communication interface circuit.

**Figure 11:**
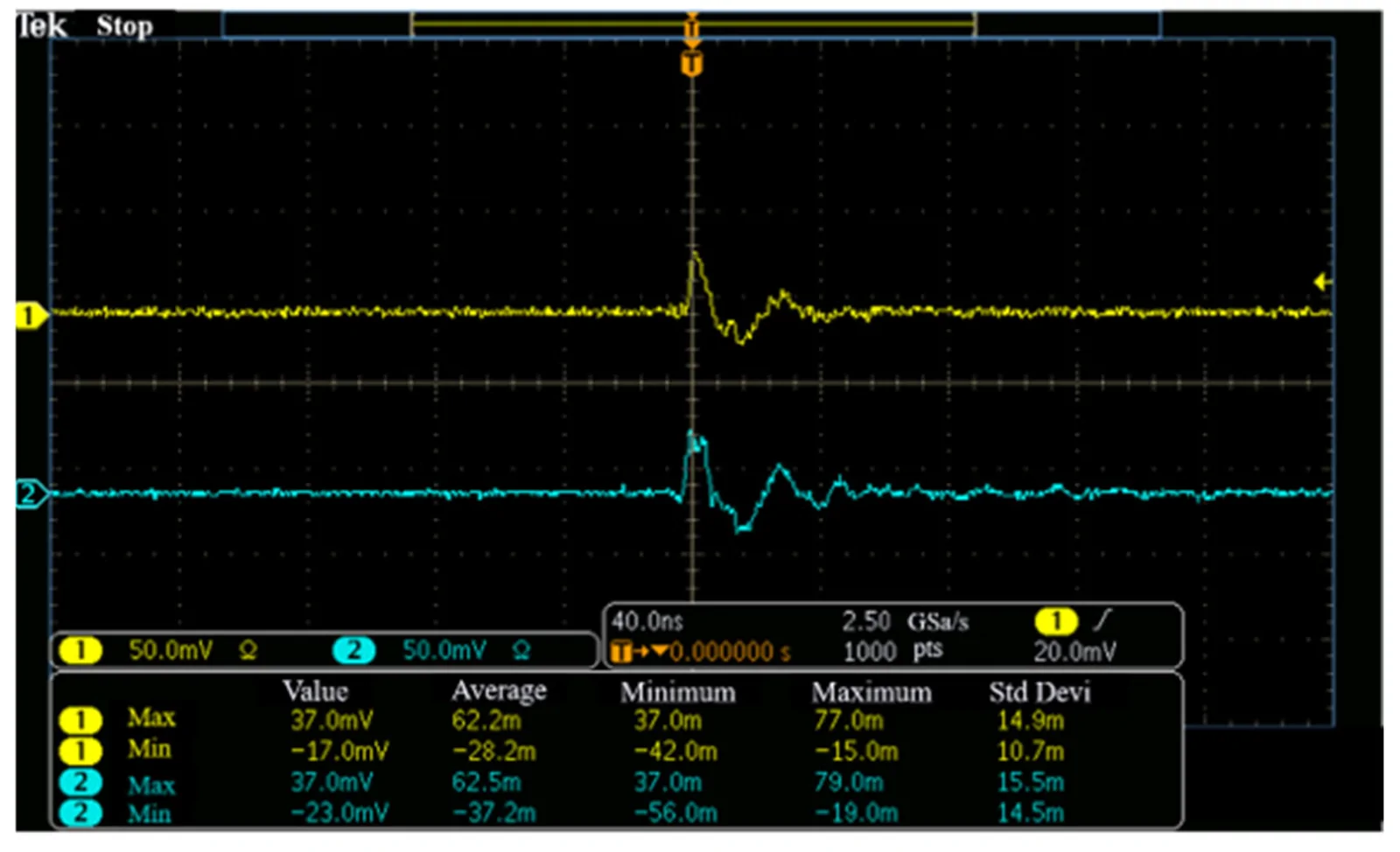
Coupling current waveform of RS-232 communication interface circuit at 500 V.

Figure 11 presents the coupling current waveform diagram of the RS-232 communication interface circuit under a source voltage of 500 V. Here, the CH1 channel of the oscilloscope is the coupling current signal of the transmitting end, and the CH2 channel is the coupling current signal of the receiving end.

From the test results shown in Table 4, when the pulse voltage set by the pulse current injection source is 3500 V, the coupling current at the transmitting end is 5.2 A and the coupling current at the receiving end is 5.36 A, and this also has a negative coupling current of about −4 A. Under this electromagnetic pulse intensity, the RS-232 communication interface circuit exhibits a transmission interruption, and the system cannot return to normal operation unless automatic recovery methods are used. Therefore, it can be determined that the interference threshold of the communication line of the RS-232 communication interface circuit is a bipolar pulse, at 5.2 A for the positive pulse and −3.1 A for the negative pulse in the emission, and 5.36 A for the positive pulse and −4.56 A for the negative pulse in the receiver.

Compared with the injection test results of the same RS-232 communication interface circuit shown in Table 5, according to the MIL-STD-461G injection test standard with a double-exponential wave injection [22,23,24,25], the peak injection voltage and coupling current values that cause damage are very different. When the attenuated oscillation wave is injected, the injection voltage that causes damage is higher than that of the double-exponential wave, and the coupling current is bipolar in waveform and significantly smaller in amplitude.

To further verify the applicability of the proposed irradiation-equivalent injection method to other low/medium-speed communication interfaces, the same injection test procedure is also carried out on the RS-485 and 100BASE-T interface circuits. For the RS-485 interface, a 63 MHz damped oscillating wave pulse is injected into the communication line between the two RS-485 interface boards, and the data transmission state is observed through the serial port debugging assistant software on Computer A and Computer B. For the 100BASE-T interface, a 26 MHz damped oscillating wave pulse is injected into the communication line between the two 100BASE-T interface boards, and the data transmission state is observed through the network port debugging assistant software. The coupling current at the transmitting end and the receiving end is measured by the current clamp, and the injection voltage is increased step by step until the data transmission cannot be recovered automatically. The test results of the RS-485 interface circuit are shown in Table 6, and the test results of the 100BASE-T interface circuit are shown in Table 7.

From Table 6 it can be seen that when the pulse voltage set by the injection source is 3000 V, the coupling current at the transmitting end of the RS-485 interface circuit is 10.48 A, and the coupling current at the receiving end is 9.92 A. Under this electromagnetic pulse intensity, the RS-485 communication interface circuit exhibits a transmission interruption, and the system cannot return to normal operation by automatic recovery methods. Therefore, the interference threshold of the communication line of the RS-485 communication interface circuit is determined to be about 9.92 A.

From Table 7, it can be seen that when the pulse voltage set by the pulse current injection source is 800 V, the coupling current at the transmitting end of the 100BASE-T interface circuit is 3.12 A, and the coupling current at the receiving end is 2.72 A. Under this electromagnetic pulse intensity, the 100BASE-T communication interface circuit exhibits a transmission interruption, and the system cannot return to normal operation by automatic recovery methods. Therefore, the interference threshold of the communication line of the 100BASE-T communication interface circuit is determined to be about 2.72 A.

It should be noted that the RS-232 interface, the RS-485 interface and the 100BASE-T interface adopt different damped oscillating wave frequencies, namely 84 MHz, 63 MHz and 26 MHz, respectively, because the coupling current frequency obtained by the field-line coupling simulation depends on the interface type and the cable length. The difference in the injection frequency reflects the difference in the coupling characteristics of different interface circuits, and also verifies that the proposed method can be applied to different types of low/medium-speed communication interfaces.

The simulation and experimental results above indicate that, in the assessment of coupling effects of typical communication interfaces and interference thresholds, the typical double-exponential pulse injection waveform is different from the coupling waveform of the interface cables during actual HEMP irradiation. Therefore, the coupling test results differ significantly from the actual irradiation situation, and are different depending on the coupling waveform and the peak value. For the normal operation of the communication interface circuit, it is necessary to design protective circuits based on accurate coupling waveforms and peak values to resist the irradiation interference from HEMP.

Although the peak values of the two coupled waveforms are not significantly different, they fall within the range that can be covered by the design parameters of the protection circuit. However, for the actual attenuation oscillation waves coupled on the cable due to irradiation, the coupling waveform on the communication interface is bipolar and has a relatively large amplitude. This needs to be given special consideration in the protection design.

## 5. Conclusions

Based on the attenuation oscillation waveforms of the port under HEMP irradiation obtained from the field-line coupling simulation, in this study we design a communication interface HEMP coupling effect test system based on real cable coupling waveforms. Taking the RS232, RS485 and 100BASE-T interfaces as the examples, the coupling currents under different injection waveforms are obtained. And according to the interface interference and failure scenarios, the interference thresholds of these interfaces for the oscillation waves are obtained. Compared with the double-exponential injection waveform in the standard, the coupling current at the time of damage is bipolar in nature and has a smaller value. This equivalent irradiation injection test method is more accurate for analyzing the coupling effect in communication interface circuits under strong electromagnetic pulse irradiation. Moreover, this method can be extended to the study of coupling effects of other interface circuits, and it has a very good reference value for the design of protective circuits.

However, it should be explicitly noted that this method is only capable of reproducing the conducted coupling effects on cables. Direct current injection cannot account for the radiative spatial effects caused by the direct illumination of the HEMP on printed circuit board assemblies or equipment enclosures. Therefore, for a comprehensive assessment of system-level HEMP susceptibility, this method should be complemented by additional full-system irradiation tests or full-wave numerical simulations that include the enclosure and PCB layouts.

## Figures and Tables

**Table 1:** Comparison between traditional test methods and the proposed scheme.

Traditional Test Methods: Problems	The Proposed Scheme: Advantages
HEMP irradiation tests require complex and costly equipment, are difficult to repeat, and have poor consistency.	The irradiation-equivalent injection method uses the simulated real coupling current waveform as the injection current, avoiding high-cost irradiation equipment, and improving repeatability and controllability.
MIL-STD-188-125 uses the double-exponential waveform as the injection basis, which differs significantly from the actual cable coupling waveform.	The proposed method uses the actual bipolar damped oscillating waveform on the cable, so the obtained failure threshold is more consistent with the real irradiation situation.
Traditional tests rely on dedicated communication facilities, lacking a general platform for wired interfaces.	A multi-interface test platform is built, which can be used to perform equivalent injection on different interfaces while ensuring normal communication.

**Table 2:** Coupling current comparison of different communication interface circuits.

Cable Length	RS-232	RS-485	100BASE-T	VGA	HDMI
/m	Freq/MHz	Freq/MHz	Freq/MHz	Freq/MHz	Freq/MHz
0.1	289	211	19	307	291
0.5	116	86	9.1	124	118
1	83	63	6.5	88	82
1.5	69	53	5.3	71	76

**Table 3:** Characteristic of the coupling waveform of the communication interface under typical working conditions.

Communication Interface Board Type	Cable Height Above Ground/m	Cable Length/m	Damped Oscillating Wave Frequency/MHz
RS-232	0.01	1	84
RS-485	0.01	1	63
100BASE-T	0.01	1	26
VGA	0.01	1	84
HDMI	0.01	1	84

**Table 4:** Test results of 84 MHz attenuated oscillation wave pulse injection of RS-232 communication interface circuit.

Injected Voltage/V	Coupled Current—Emission (Max CH1)	Coupled Current—Emission (Min CH1)	Coupled Current—Receiver (Max CH2)	Coupled Current—Receiver (MinH2)	Interface Data Transmission
500	0.74	−0.34	0.74	−0.46	Normal Data Transmission
700	1.06	−0.66	1.14	−0.70	Normal Data Transmission
……	……	……	……	……	……
3300	4.72	−2.80	4.72	−4.08	Normal Data Transmission
3500	5.20	−3.12	5.36	−4.56	Transmission Interruption, Unable to Recover Automatically, Recoverable After Restart

**Table 5:** Test result of direct injection of HEMP source of RS-232 interface circuit.

Injected Voltage (V)	Peak Coupled Current (A)	Interface Data Transmission
500	8.04	Normal Transmission
1000	15.9	Normal Transmission
1100	16.5	Symbol Waveform Output Degradation
1200	18.7	Transmission Interruption

**Table 6:** Test results of 63 MHz attenuated oscillation wave pulse injection of RS-485 communication interface circuit.

Injected Voltage/V	Coupled Current—Emission (Max CH1)	Coupled Current—Emission (Min CH1)	Coupled Current—Receiver (Max CH2)	Coupled Current—Receiver (MinH2)	Interface Data Transmission
500	1.70	−1.14	1.62	−1.34	Normal Data Transmission
700	2.42	−1.34	2.30	−1.34	Normal Data Transmission
……	……	……	……	……	……
2900	10.00	−6.00	9.60	−5.92	Normal Data Transmission
3000	10.48	−6.16	9.92	−6.08	Transmission Interruption, Unable to Recover Automatically, Recoverable After Restart

**Table 7:** Test results of 26 MHz attenuated oscillation wave pulse injection of 100BASE-T communication interface circuit.

Injected Voltage/V	Coupled Current—Emission (Max CH1)	Coupled Current—Emission (Min CH1)	Coupled Current—Receiver (Max CH2)	Coupled Current—Receiver (MinH2)	Interface Data Transmission
300	1.08	−0.20	0.92	−0.32	Normal Data Transmission
400	1.44	−0.28	1.16	−0.44	Normal Data Transmission
……	……	……	……	……	……
700	2.40	−1.12	2.00	−1.12	Normal Data Transmission
800	3.12	−0.32	2.72	−0.72	Transmission Interruption, Unable to Recover Automatically, Recoverable After Restart

## Data Availability

The data used to support the findings of this study are available from the corresponding author upon request.

## Abbreviations

The following abbreviations are used in this manuscript:

HEMP	High-altitude nuclear electromagnetic pulse
AC	Alternating Current
HV	High-Voltage
SMPS	Switched-mode power supply
ECU	Electronic Control Unit
